# The COVID-19 Applicant: The Rise of Twitter Among Matched Neurosurgery Applicants

**DOI:** 10.7759/cureus.46383

**Published:** 2023-10-02

**Authors:** Bao Y Sciscent, Cara E Pearson, Casey Ryan, Lekhaj C Daggubati

**Affiliations:** 1 Medicine, Penn State Health Milton S. Hershey Medical Center, Hershey, USA; 2 Neurosurgery and Brain Repair, University of South Florida, Tampa, USA; 3 Neurosurgery, Penn State Health Milton S. Hershey Medical Center, Hershey, USA

**Keywords:** match, residency, social media, covid-19, twitter

## Abstract

Introduction: Social media is becoming increasingly ubiquitous in the professional realm. The coronavirus disease 2019 (COVID-19) pandemic accelerated the shift towards utilizing social media to network and disseminate information, especially via Twitter. Neurosurgery programs have also leveraged Twitter to inform and attract applicants.

Objective: The purpose of this study is to identify factors influencing the adoption of Twitter by matched neurosurgery applicants before and during the COVID-19 pandemic.

Methods: A list of matched U.S. neurosurgery residents from just before the start of the pandemic (2019-2020) to after the peak of the pandemic (2021-2022), was collated. Twitter was searched to evaluate the presence of a professional account at the time of Electronic Residency Application Service (ERAS) submission. The following demographic variables were collected: gender, medical school, and matched residency institution.

Results: Over four application cycles (2019-2022), 897 matched residents were evaluated in the study. Overall, 31.1% had a Twitter account during the time of their residency application. In particular, international medical school graduates were more likely to have a Twitter platform compared to U.S. applicants (50.0% vs. 29.7%; *p=.*001). The percentage of matched neurosurgery applicants with a Twitter profile significantly increased during the pandemic (21.0% vs. 41.1%; *p<.*001) with a two-fold increase from 20.0% to 39.7% (*p<*.001) in U.S. applicants.

Conclusion: Over the past four years, an increasing number of matched neurosurgery applicants have adopted a Twitter presence during application. Driven by the increasing use of social media and accelerated by the COVID-19 pandemic, Twitter has become an important tool leveraged by during the application process.

## Introduction

Over the last few years in the setting of the coronavirus disease 2019 (COVID-19) pandemic, there has been a noticeable resurgence of the social media app Twitter, and this revival seems to be a repurposing of the application into the professional realm as a news and networking platform more than ever before. This resurgence has expanded into the medical field, where there has been a wide and unanimous increase in its usage by residency applicants and residency programs alike, which seems to correlate with the timing of a new global focus on a more remotely accessible world that surfaced during the COVID-19 pandemic [1].

Limited in-person opportunities and virtual interviews have encouraged applicants and programs alike to adapt to using social media platforms like Twitter to form professional connections, share medical information, and broadcast personal and departmental updates [2,3]. Competitive specialties, such as neurological surgery, have historically emphasized away rotations and sub-internships for one’s application [4,5]. Additionally, medical schools have shifted their focus more towards subjective rather than objective measures for the evaluation of applicants with more schools having a pass/fail curriculum, emphasizing research productivity, and clinical assessments from third- and fourth-year rotations [6]. With the transition to a nearly completely virtual experience starting with the 2021 cycle due to the implications of the COVID-19 pandemic, applicants had to navigate new platforms to increase their visibility and competitiveness. The purpose of this study is to identify trends and factors influencing the adoption of the Twitter platform by matched neurosurgery applicants during the COVID-19 pandemic.

This article was previously presented as a meeting virtual poster abstract at the 2021 CNS Meeting on October 16-20, 2021.

## Materials and methods

Publicly available information for U.S. neurosurgery residency programs and matched applicant names from 2019 to 2022 was obtained through the Association of Neurological Surgeons (AANS) and the respective program websites of the institutions that matched residents [7]. Applicant names were searched on Twitter for an active professional account. Accounts that were easily identifiable and exhibited some level of engagement with neurosurgery, either by following neurosurgery department accounts or publishing posts about neurosurgery, were analyzed. Account creation dates were recorded and then compared against deadlines for the Electronic Residency Application Service (ERAS) for each year. Demographic information was collected on each matched applicant’s gender, medical school, presence of an international medical education (IMG), and matched residency program.

Descriptive statistics, including frequencies and percentages, were used to report demographic information. A Chi-squared test of independence was performed in IBM SPSS (IBM Corp., Armonk, NY, USA) to determine if there were significant associations between the presence of a Twitter account and gender, medical school, presence of IMG medical education, match year, and matched institution. An additional subgroup analysis was performed to determine if there was any relationship between the presence of applicants having a Twitter account prior to ERAS submission with the timing of the COVID-19 pandemic by stratifying the data based on the demographic factors of either gender, IMG status, or home match. A p-value <0.05, representing a 95% confidence interval, was considered statistically significant.

## Results

A total of 897 matched residents were evaluated between the years of 2019 to 2022. There were 222, 225, 231, and 219 applicants in the years 2019, 2020, 2021, and 2022, respectively. Applicants were split into groups based on whether they matched before or during the pandemic. Those who matched in 2019 and 2020 were considered to have matched pre-pandemic, since ERAS submission deadlines and conclusion of interviews occurred before March 2020. Those who matched in 2021 and 2022 were in the pandemic group.

Across all years, 31.1% (n=279) of matched applicants had a Twitter account before ERAS submission. Of 897 matched applicants, 658 (73.4%) were male and 239 (26.6%) were female. A total of 837 (93.3%) applicants graduated from a U.S. medical school and 60 (6.7%) graduated from an international medical school. About one-fifth (n=180, 20.1%) of applicants matched at their home institution. The majority (n=717, 79.9%) of students matched at another institution outside of their home medical education program. Across all neurosurgery applicants over the four application years observed in the present study, 279 (31.1%) had a Twitter presence prior to ERAS. These demographics are listed in Table 1. 

**Table 1:** Demographics of Matched Neurosurgery Applicants between 2019-2022 IMG: international medical graduates ERAS: electronic residency application service

Demographics		Applicants, n (%)
Gender	Female	239 (26.6%)
	Male	658 (73.4%)
Medical Education	U.S. Graduates	837 (93.3%)
	IMG	60 (6.7%)
Home institution match	Home institution	180 (20.1%)
	Outside institution	717 (79.9%)
Twitter Prior to ERAS	Yes	279 (31.1%)
	No	618 (68.9%)

Across all applicants, 207 (31.5%) males and 72 (30.1%) females had a professional Twitter profile, although there was no significant difference between gender and the presence of an active account (p=.703). IMGs made up 6.7% of all matched residents, but were more likely to have a Twitter profile compared to U.S. graduates (50.0% vs. 29.7%; p=.001). There was no significant difference between the percentage of applicants with a Twitter profile who matched and those who did not match at their home institution (30.6% vs. 31.2%; p=.859). Between the pre-pandemic and pandemic groups, the percentage of matched applicants with an account nearly doubled (21.0% vs. 41.1%; p<.001). Only 14.9% of applicants had a Twitter in 2019, compared to 40.2% in 2022 (p<.001). These results are listed in Table 2.

**Table 2:** Analyses of Matched Neurosurgery Applicants with a Twitter prior to ERAS Bolded values are significant IMG: international medical graduates ERAS: electronic residency application service

		Twitter Presence Prior to ERAS Submission		
Variable		Yes, n (%)	No, n (%)	p-value
Total	2019-2022	279 (31.1%)	618 (68.9%)	
Gender	Male	207 (31.5%)	451 (68.5%)	.703
	Female	72 (30.1%)	167 (69.9%)	
Home institution match	Yes	55 (30.6%)	125 (69.4%)	.859
	No	224 (31.2%)	493 (68.8%)	
Country of Education	IMG	30 (50.0%)	30 (50.0%)	.001
	U.S. Graduates	249 (29.7%)	588 (70.3%)	
Year	2019	33 (14.9%)	189 (85.1%)	<.001
	2020	61 (27.1%)	164 (72.9%)	
	2021	97 (42.0%)	134 (58.0%)	
	2022	88 (40.2%)	131 (59.8%)	
Pandemic	Pre-pandemic	94 (21.0%)	353 (79.0%)	<.001
	Pandemic	185 (41.1%)	265 (58.9%)	

Sub-group analyses were performed to compare differences in matched applicants pre-pandemic and during the pandemic while focusing on variables such as gender, country, and home program match which are shown in Table 3.

**Table 3:** Sub-group analysis of Matched Neurosurgery Applicants with a Twitter prior to ERAS before and during the pandemic Bolded values are significant IMG: international medical graduates ERAS: electronic residency application service

Variable		Twitter Presence Prior to ERAS Submission		
Gender		Yes, n (%)	No, n (%)	p-value
Female	Pre-Pandemic	27 (22.9%)	91 (77.1%)	.016
	Pandemic	45 (37.2%)	76 (62.8%)	
Male	Pre-Pandemic	67 (20.4%)	262 (79.6%)	<.001
	Pandemic	140 (42.6%)	189 (57.4%)	
Country of Education				
U.S.	Pre-Pandemic	84 (20.0%)	337 (80.0%)	<.001
	Pandemic	165 (39.7%)	251 (60.3%)	
IMG	Pre-Pandemic	10 (38.5%)	16 (61.5%)	.118
	Pandemic	20 (58.8%)	14 (41.2%)	
Home Match				
Home	Pre-Pandemic	22 (23.9%)	70 (76.1%)	.048
	Pandemic	33 (37.5%)	55 (62.5%)	
Outside institution	Pre-Pandemic	72 (20.3%	283 (79.7%)	<.001
	Pandemic	152 (42.0%)	210 (58.0%)	

The number of male applicants with a professional Twitter profile significantly increased from 67 (20.4%) pre-pandemic to 140 (42.6%) during the pandemic (p<.001). A similar, sizeable increase was also seen in female users from 27 (22.9%) pre-pandemic to 45 (37.2%) during the pandemic (p=.016). During the pandemic, there was a statistically significant increase in the number of U.S. medical school graduates who adopted a Twitter platform (20.0% vs. 39.7%; p<.001). The percentage of IMGs with an account increased from 38.5% to 58.8%, but this did not account for a statistically significant increase (p=.118). The number of applicants with a Twitter account who matched at their home institution significantly increased during the pandemic (24.4% vs. 37.5%; p=.048). A significant increase was also seen in those who matched outside of their home institution from 20.3% of applicants with a Twitter pre-pandemic to 42.0% during the pandemic (p<.001).

## Discussion

Over the last few years, the COVID-19 pandemic has stimulated the growth of a social media presence among residency applicants [3]. The rise of academic social media use among neurosurgeons and neurosurgical departments allows for increased opportunities for advocacy and networking [1]. It provides a real-time platform to instantly broadcast one’s unique narrative, which can be advantageous for applicants. Prior studies have demonstrated substantial growth in residency programs using social media platforms to find new ways of promoting their program and attracting applicants [2,8,9]. Among neurosurgery departments, there was a 130% rise in the number of Twitter accounts created between 2019-2021 [1].

This study investigated trends in the use of Twitter by matched neurosurgery applicants before and during the pandemic. This study demonstrates that the percentage of matched neurosurgery applicants with a Twitter account rose from 21.0% to 41.1% during the pandemic, particularly U.S. graduates who seemed to adopt the trend. One potential reason for the growth of social media during a period of national isolation and mitigation of large social events could be the small size and number of neurosurgeons and neurosurgery departments compared to other medical specialties. Given that the community tends to be tight-knit due to its compact size, there is an increasing emphasis on the value of networking and being linked into the field. With the transition to remote interview seasons and the inability to interact face-to-face with residents and faculty, the real-time social media application may have served as the best substitute for in-person insight into programs.

The lack of in-person evaluations through sub-internships (Sub-Is) and interviews due to the pandemic impacted an applicant’s view of an institution as well as the converse, the program’s ability to assess an applicant [10]. Neurosurgery, like most competitive surgical specialties, has a high rate of matching applicants who did a Sub-I at their institution [11]. These Sub-Is allow applicants to display their abilities, acquire letters of recommendation, and build relationships at outside institutions. Our study showed a significant increase in the number of matched applicants with a Twitter profile who matched at an outside institution, but also in those who matched at their home department during the pandemic compared to prior. Although there are many other variables probably at play regarding the demographics of the match statistics after the start of the pandemic and the beginning of a remote interview process, it is interesting to see that the presence of a career-focused social media platform was significantly associated with a changing matching profile. The significance of social media can also be seen in our observation of a larger Twitter presence of matched IMGs overall and prior to the pandemic, compared to U.S. graduates. These applicants may not have had the same advantages of in-person rotations and may have used Twitter to increase visibility and engage with programs.

At the same time, residency applicants across multiple disciplines have reported using social media websites to gain information about a program’s culture, stay informed about event dates, and learn about residents’ social life [12-14]. In particular, Twitter offers a unique platform for networking compared to other social media outlets because it allows users to showcase their achievements, follow program updates, discover educational resources, and connect with other applicants [3,15]. Some Twitter users were even able to attend virtual sub-internships during the 2021 application cycle [8]. Although these opportunities are not equivalent to in-person experiences, they allow for informal interaction and continued medical education in a competitive specialty.

Although individuals usually manage their own Twitter accounts, our study is limited and dependent on the accuracy of online information, which may potentially be inaccurate. This study only utilized publicly available data, which led to some missing data points that could not be found online. Additionally, factors outside of the pandemic that may have encouraged applicants to adopt Twitter during this period were not studied. Furthermore, although we only included professional Twitter accounts, the presence of an account does not equal utility. Lastly, the focus of this study is Twitter use during the COVID-19 pandemic, so this only represents data from a four-year sample.

## Conclusions

Twitter is a powerful tool because it is a free resource and allows for the instant showcasing of information and communication between users. Increased utility and awareness of social media platforms over the last few years is reflected in the increased percentage of matched neurosurgery applicants with Twitter, particularly U.S. graduates. Further prospective surveys and retrospective analyses are still needed to quantify the role of social media for program directors, matched applicants, and unmatched applicants. As COVID-19 restrictions are being lifted and neurosurgery programs are returning to in-person events, it will be interesting the monitor if programs and applicants continue to maintain and promote their Twitter presence. Future studies should also monitor this trend in other specialties where sub-internships and other in-person networking events are not as strongly emphasized. Twitter is an increasingly popular platform for neurosurgery applicants to leverage throughout the application cycle. The COVID-19 pandemic has seen an increasing number of matched neurosurgery applicants on Twitter at the time of application.
